# Community-associated Methicillin-resistant *Staphylococcus aureus* Isolates and Healthcare-Associated Infections^1^

**DOI:** 10.3201/eid1302.060781

**Published:** 2007-02

**Authors:** Cynthia L. Maree, Robert S. Daum, Susan Boyle-Vavra, Kelli Matayoshi, Loren G. Miller

**Affiliations:** * David Geffen School of Medicine at the University of California, Los Angeles, California, USA; † University of Chicago, Chicago, Illinois, USA; ‡ Los Angeles Biomedical Institute at Harbor-UCLA Medical Center, Torrance, California, USA

**Keywords:** Community associated-MRSA, healthcare-associated infections, MRSA, research

## Abstract

We noted a marked increase in healthcare-associated (HA) methicillin-resistant *Staphylococcus aureus* (MRSA) infections caused by isolates phenotypically consistent with community-associated (CA)-MRSA strains. To study this trend, we retrospectively examined all HA-MRSA isolates from patients in our institution during 1999–2004. An isolate was considered an SCC*mec*IV phenotype if it had antimicrobial drug susceptibilities consistent with typical CA-MRSA isolates. Our phenotypic definition was validated in a limited subset of isolates by SCC*mec* genotype, pulsed-field gel electrophoresis, and multilocus sequence typing. Among 352 patients with HA-MRSA isolates, SCC*mec*IV phenotype increased from 17% in 1999 to 56% in 2003 (p<0.0001). Antimicrobial drug-susceptibility phenotype and genotype were consistent in 21 (91%) of 23 isolates. In a multivariate model, the SCC*mec* type IV phenotype was independently associated with wound culture source, later year of collection, and MRSA isolated earlier during hospitalization. In conclusion, MRSA isolates phenotypically similar to CA strains have become the predominant isolates associated with HA-MRSA in our hospital.

## Abstract 2

MRSA isolates phenotypically similar to community-associated strains have become the predominant isolates associated with healthcare-associated MRSA in our hospital.

Methicillin-resistant *Staphylococcus aureus* (MRSA) is the most frequently identified antimicrobial drug–resistant pathogen in US hospitals (*1*). The epidemiology of infections caused by MRSA is rapidly changing. In the past 10 years, infections caused by this organism have emerged in the community. The 2 MRSA clones in the United States most closely associated with community outbreaks, USA400 (MW2 strain, ST1 lineage) and USA300, often contain *pvl* genes and, more frequently, have been associated with skin and soft tissue infections (*2*,*3*). Outbreaks of community-associated (CA)–MRSA infections have been reported in correctional facilities, among athletic teams, among military recruits, in newborn nurseries, and among men who have sex with men (*4*–*7*). CA-MRSA infections now appear to be endemic in many urban regions and cause most CA–*S. aureus* infections (*5*,*6*,*8*–*10*).

CA-MRSA isolates were first recognized by distinct resistance profiles of antimicrobial drugs that lacked resistance to older antimicrobial drugs (*11*–*13*). Several groups have noted these distinct susceptibility patterns appearing in isolates from hospitalized patients. Denis et al. noted that since 1995, MRSA isolates in Belgian hospitals were losing resistance to older antimicrobial drugs such as gentamicin and clindamycin (*14*). A Spanish hospital experienced a decrease in gentamicin-resistant MRSA isolates (from 97% in 1998 to 20% in 2002) and a simultaneous increase in MRSA isolates carrying the SCC*mec* type IV cassette (from 0% prevalence in 2000 to 23% prevalence in 2002) (*15*). A French group noted a similar finding in their hospitals over an 11-year period and found a correlation between isolates that contained SCC*mec* type IV and susceptibility profiles to ≥3 antimicrobial drugs (*16*). However, these investigations did not distinguish between cultures obtained from patients hospitalized with CA infection and those with hospital-associated (HA) infections. Thus, it is unclear whether these trends in decreased antimicrobial drug resistance and increased number of MRSA isolates that contained SCC*mec* type IV were due to increased hospitalization of patients with CA-MRSA infections or to an increased prevalence of isolates containing SCC*mec* type IV among HA-MRSA isolates.

Some MRSA strains associated with CA infection have been noted to cause HA infections. Outbreaks of HA infections caused by isolates containing SCC*mec* type IV have been reported from Australia and the United States. Affected populations have included postpartum women and patients undergoing prosthetic joint replacement (*17*–*19*). Another recent report demonstrated that CA strains had emerged as a substantial cause of HA bloodstream infections (*20*). However, these reports are anecdotal, and data examining temporal trends are lacking.

At our institution, which is located in an area in which CA-MRSA infections are endemic, we have noted a large increase in HA infections caused by MRSA isolates that, by assessment of antibiotic susceptibility patterns, appear to carry the SCC*mec* type IV element (e.g., susceptible to gentamicin, clindamycin, and trimethoprim-sulfamethoxazole) (*6*,*10*,*21*). The aim of this study was to quantify this trend over a 6-year period.

## Methods

### Population

To find patients with HA-MRSA infections, we identified all cultures obtained >72 hours after hospitalization that grew MRSA, from January 1, 1999, through December 31, 2004, at Harbor-UCLA Medical Center, a tertiary-care, urban, county hospital in Los Angeles County. At this hospital, surveillance cultures for MRSA colonization are not routinely performed; therefore, cultures positive for MRSA are likely to reflect infection rather than colonization. For a given patient, we examined only data from the first positive culture and excluded patients who had positive cultures both ≥72 hours and <72 hours after admission. If a patient had been hospitalized more than once during the study period, only data from the first hospitalization were retained. A standardized instrument was used to abstract data from the medical record of each patient. Information obtained included demographics, admission date and time, hospital location, antimicrobial drug susceptibility of the MRSA isolate, and time, date, and source of the MRSA culture.

We obtained only MRSA blood isolates for molecular typing because the clinical microbiology laboratory discards all other types of isolates after identification is complete. In vitro susceptibilities were reported as minimal inhibitory concentrations and performed with the VITEK system (bioMérieux, Durham, NC, USA), according to the protocols of the Clinical and Laboratory Standards Institute (CLSI). The investigation protocol was reviewed and approved by the Institutional Review Board of Harbor-UCLA Medical Center.

### Molecular Characterization of Strains

Molecular typing was performed at the University of Chicago by investigators who were blinded to the clinical details and antibiograms of the isolates.

### SCC*mec* Typing

PCR was performed to detect *mecA* by using the primer pair mecAF/mecAR (*22*). SCC*mec* elements were distinguished by the molecular architecture of the *ccr* and *mecA* complexes (*21*,*23*,*24*). PCR typing of SCC*mec* types I–IV was performed under the conditions previously described (*24*,*25*). SCC*mec* type II (*ccrAB* complex type 2 and *mec* complex class A), SCC*mec* type III (*ccrAB* complex type 3 and *mec* complex class A), and SCC*mec* type IV (*ccrAB* complex type 2 and *mec* complex class B) were assigned according to published criteria (*25*). PCR primers used to detect *mecI* (primers mI3/mI4), the *mecR1* membrane spanning region (MS) (primers mcR3/mcR4), and the *mecR1* penicillin-binding region (PB) (primers mcR1/mcR5) were originally reported by Suzuki et al. (*26*). Screening for *ccrAB* complex types 1, 2, and 3 (*ccrAB* 1, 2 and 3) was accomplished with a multiplex PCR assay that uses a mixture of 4 primers designed by Ito et al., consisting of a common forward primer (β2) and reverse primers, α2, α3, and α4 specific for *ccrAB* complexes 1, 2, and 3. Thermocycler conditions used have been described (*27*). The presence of the *ccrAB* gene complex allotype 4 (*ccr complex 4*) was assessed in a separate reaction that used the primer pair ccrA4F and ccrB4R (*27*). Screening for the *ccrC* gene (*ccr complex 5*) was performed with a forward primer (γF) in combination with the reverse primer γR described by Ito et al. (*28*). Prototype strains used for SCC*mec* typing were NCTC10442 (SCC*mec* I), N315 (SCC*mec* II), 85/2082 (SCC*mec* III), MW2 (SCC*mec* IV), and WIS (SCC*mec* V). The control strain used for detection of *ccrAB4* was *S. epidermidis* strain ATCC 12228, which contains *ccrAB4* in the non–*mec-*containing SCCcomposite island (*24*).

### MLST

MLST was performed by PCR amplification and sequencing of 7 housekeeping genes by using the primer pairs designed by Enright et al (*29*). Denville Taq-Pro Complete (Denville Scientific, Metuchen, NJ, USA) or the Taq DNA Polymerase (Promega, Madison, WI, USA) was used for the PCR reactions. PCR products were evaluated on an agarose gel and purified by using Millipore 96-well Montage (Billerica, MA, USA) plates according to manufacturer’s instructions. The purified templates were sequenced at the University of Chicago Core Sequencing Facility and evaluated with the use of Vector NTI software (Invitrogen, Carlsbad, CA, USA). Each sequence was submitted to the MLST database website (www.mlst.net) for assignment of the allelic profile and sequence type (ST).

### Screening for *pvl* Genes

Isolates were screened for the *lukF-PV* and *lukS-PV* genes encoding the components of the PVL toxin by PCR amplification of a 433-bp product that includes a portion of both the *lukS-PV* and *lukF-PV* ORFs by using the primer pair luk-PV-1/ luk-PV-2 (final concentration 0.2 μM) designed by Lina et al. (*30*). The thermocycler conditions have been described (*27*).

### Case Definition and Data Analysis

A standardized definition of CA-MRSA infection was created by the Centers for Disease Control and Prevention (CDC) Active Bacterial Core Surveillance sites (*31*). Using this definition, we defined HA-MRSA infections as those MRSA infections that did not meet the definition of CA-MRSA infections. Specifically, we defined an MRSA isolate as HA associated if the original entry criteria of hospitalization for >72 hours before culture acquisition was met and if in the year before the present hospitalization, the patient had had any 1 of the following: hospitalization, surgery, residency in a long-term care facility, and hemodialysis or peritoneal dialysis, or at the present admission had indwelling percutaneous devices or catheters. A CA infection was defined as a culture-confirmed MRSA infection without any of the above criteria. However, if the patient did not meet any of the above criteria, had an infection at the time of admission, and the culture of the infection on admission was taken ≥72 hours after admission, then the infection was considered CA. An example of this situation would be a deep tissue infection microbiologically diagnosed from a surgical biopsy specimen 4 days after the patient’s admission.

To validate our definition of HA-associated infection, we reviewed 105 (30%) randomly selected charts of the patients with MRSA infections identified ≥72 hours after hospitalization. The purpose of this validation was to confirm that these cultures did not reflect CA infections that were diagnosed late (>72 hours) in the hospital course. Of note, in the CDC definition, an infection is considered HA if it occurs >48 hours after admission. Yet, we chose >72 hours as a cut-off to more conservatively capture HA infections, i.e., to minimize the miscategorization of CA infections as HA infections.

We then defined MRSA strains as having the SCC*mec* type IV phenotype if the isolates were resistant to oxacillin and susceptible to gentamicin, clindamycin, and trimethoprim-sulfamethoxazole. All other isolates were considered to be phenotypically non–SCC*mec* type IV.

Characteristics were compared between patients infected with the non–SCC*mec* type IV phenotype isolates and those infected with SCC*mec* type IV phenotype isolates by using a χ^2^ or *t* test, as appropriate. No adjustments were made for multiple comparisons. Temporal trends in the proportion of the SCC*mec* type IV phenotype were compared with the Cochran-Armitage test of trends. A multivariate analysis that predicted phenotypically SCC*mec* type IV isolates was performed by using an unconditional logistic regression model and a backward model selection method. A p value of <0.05 was defined as statistically significant. Data analysis was done with SAS software version 8.2 (SAS Institute Inc., Cary, NC, USA).

## Results

### Population Characteristics

We identified 352 patients who had HA-MRSA cultures; 229 (65%) were men, and the median age was 50 years (mean 49.5 years). In the subset of medical records reviewed for validation of HA or CA status, none of the patients’ infections (0/105) fit our definition of a CA infection. The SCC*mec* type IV phenotype was identified in 128 (36%) of these 352 patients. Compared with the non–SCC*mec* type IV phenotype, patients with the SCC*mec* type IV phenotype were younger (median age 48 vs. 54 years, p = 0.02) and had the defining culture taken earlier in the hospitalization (median 8 vs. 15 days, p = 0.01). Finding an isolate with the SCC*mec* type IV phenotype was more likely if the culture source was from a wound, blood, or source other than sputum (odds ratio [OR] 2.9, 95% confidence interval [CI] 1.7–5.0, p<0.0001; OR 2.6, 95% CI 1.2–5.7, p = 0.02; and OR 1.2, 95% CI 0.6–2.3, p = 0.69) (Table 1).

**Table 1:** Patients with healthcare-associated MRSA isolates, 1999–2004, and predictors of SCCmec type IV phenotype*

Characteristic	Patients with HA-MRSA Isolates, % (no.)	SCC*mec* phenotype,% (no.)		Predictors of SCC*mec* type IV phenotype			
				Bivariate anaylsis		Multivariate analysis	
		Type IV	Type II/III	Odds ratio (95% CI)	p value	Odds ratio (95% CI)	p value
Total	(352)	36 (128)	64 (224)				
Sex							
F	35 (123)	36 (44)	64 (79)		0.87		>0.05
M	65 (229)	37 (84)	63 (145)				
Age, y							
Mean	50 ± 19 SD	47±1.7 SE	51±1.3 SE		**0.02**		>0.05
Median (range)	50 (<1–97)	48 (<1–87)	54 (<1–97)				
Time from admission to specimen collection, d							
Mean	19 ± 21 SD	15±1.9 SE	21±1.4 SE		**0.01**	0.88 (0.8–0.98)†	**0.02**
Median (range)	12 (4–184)	8 (4–174)	15 (4–184)				
Culture specimen source							
Blood	10 (35)	46 (16)	54 (19)	2.6 (1.2–5.7)	**0.02**	2.2 (0.97–5.0)	0.058
Sputum	34 (188)	25 (29)	75 (89)	Reference		Reference	
Wound	38 (133)	49 (65)	51 (68)	2.9 (1.7–5.0)	**<0.0001**	2.6 (1.5–4.6)	**0.001**
Other	19 (66)	27 (18)	73 (48)	1.2 (0.6–2.3)	0.69	1.1 (0.5–2.2)	0.82
Year culture specimen collected							
1999	52 (15)	17 (9)	83 (43)	Reference	**<0.0001‡**	Reference	**<0.0001**
2000	57 (16)	19 (11)	81 (46)	1.1 (0.4–3.0)		NS§	
2001	65 (19)	28 (18)	72 (47)	1.8 (0.7–4.5)		1.8 (0.9–3.7)	
2002	47 (13)	43 (20)	57 (27)	3.5 (1.4–8.9]		3.2 (1.5–6.9)	
2003	63 (18)	56 (35)	44 (28)	6.0 (2.4–14.3)		5.2 (2.5–10.5)	
2004	68 (19)	52 (35)	48(33)	5.1 (2.1–12)		4.4 (2.2–8.8)	

*MRSA, methicillin-resistant *Staphylococcus aureus*; HA, healthcare associated; CI, confidence interval; SD, standard deviation; SE, standard error; NS, not significant; **boldface** indicates significance. †Multivariate analysis results reflect time in weeks. ‡Measured with the Cochran-Armitage test of trends. §Was not a significant predictor in the multivariate model.

### Validation of the SCC*mec* Phenotype Definition

Of the 352 cultures, 35 were recovered from blood and were potentially available for genetic analysis. We were able to subculture 24 of the blood isolates. We could not perform SCC*mec* typing on 1 of the 24 growing isolates. The 23 remaining isolates were representative of each year of the 6-year period except 1999, when no isolates could be recovered.

Twelve isolates carried the SCC*mec* type IV element, and 9 also carried the *pvl* genes (Table 2). Eleven isolates carried the SCC*mec* type II element; none carried *pvl*. The clinical definition of the SCC*mec* IV phenotype was fulfilled by 11 (92%) of the 12 isolates that carried the SCC*mec* IV element. The exception was an isolate that contained SCC*mec* IV that was resistant to gentamicin, clindamycin, and trimethoprim-sulfamethoxozole. The definition of the non-SCC*mec* IV phenotype was fulfilled by 10 (91%) of 11 isolates carrying the SCC*mec* II element. Phenotypic case definition of SCC*mec* type was highly correlated with the genotype confirmation of the SCC*mec* type phenotype (p<0.0001 by Fisher exact test).

**Table 2:** Summary of genetic testing for 24 healthcare-associated MRSA blood isolates*†

No. isolates	SCC*mec*	*mec*I	*mecR1* (PB)	**pvl**	MLST	Clindamycin	Gentamicin	TMP-SMX	SCC*mec* type phenotype‡
8	IV	–	–	+	8	S	S	S	IV
1	IV	–	–	+	1	S	S	S	IV
2	IV	–	–	–	8	S	S	S	IV
1*	IV	–	-	–	8	R	R	R	II
5	II	+	+	–	5	R	R	S	II
4	II	+	+	–	5	R	S	S	II
1	II	+	+	–	8	R	S	S	II
1§	II	+	+	–	5	S	S	S	IV
1¶	-	–	–	–	5	R	S	S	II

*MRSA, methicillin-resistant *Staphylococcus aureus*; PB, penicillin-binding region;;MLST, multilocus sequence typing; R, resistant; S, susceptible; TMP-SMX, trimethoprim-sulfamethoxazole. †All isolates were associated with *mecA* and *ccr2* genes. ‡Phenotype: as defined by the study case definition, according to antimicrobial drug susceptibilities (see Methods). §SCC*mec* phenotype did not match genotype. ¶SCC*mec* was not located for this isolate.

### Trend and Multivariate Analysis of the SCC*mec* type IV Phenotype

The proportion of MRSA isolates with the SCC*mec* type IV phenotype increased from 17% in 1999 to 56% in 2003 (p<0.0001, Figure). The proportion of isolates that were of the SCC*mec* type IV phenotype in 2004 (52%) was little changed from 2003 (Figure). In the multivariate model, independent predictors for having an SCC*mec* type IV phenotype isolate were wound source of culture (referent group was sputum source, OR 2.6, 95% CI 1.5–4.6, p = 0.001), culture obtained in less time after admission, (OR 0.88 per week, 95% CI 0.8–0.98, p = 0.02), and year of culture acquisition (p<0.0001) (Table 1).

**Figure:**
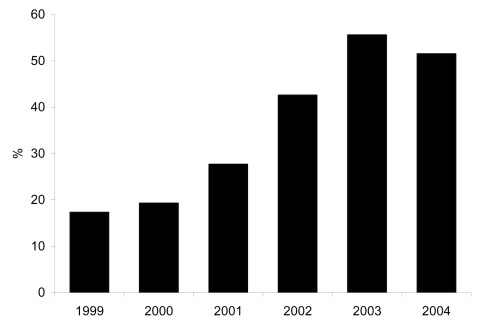
Percentage of methicillin-resistant Staphylococcus isolates that are SCC*mec* type IV phenotype, 1999–2004

## Discussion

In many urban centers worldwide, infections due to MRSA account for a large proportion of CA–*S. aureus* infections; in some communities MRSA accounts for more than half of CA–*S. aureus* infections (*6*,*8*–*10*,*32*). There have been reports of strains frequently associated with community outbreaks causing HA infections, but they have been mostly limited to case reports or case series (*17*–*19*). To our knowledge, ours is the first investigation quantifying the rise of MRSA isolates typical of CA disease to become the predominant strain of HA-MRSA (i.e., accounting for >50% of MRSA strains) within the hospital setting. Remarkably, at our institution the number of HA-MRSA isolates that have a CA phenotype, which previously was uncommon, now is >50%.

Our analysis found 3 significant risk factors for an SCC*mec* type IV phenotype MRSA culture. First, patients with MRSA cultures from a wound source were more likely to have the SCC*mec* type IV phenotype. This finding may be understandable, given that the most common clinical syndrome described with CA-MRSA infections has been skin and soft tissue infections (*10*,*33*). In addition, 75% of CA-MRSA isolates that were genotyped carried the *pvl* gene, which has a strong association with skin and soft tissue infections (*33*). A second risk factor for the SCC*mec* type IV phenotype was a shorter length of hospital stay before MRSA culture. This association may be due to the increased severity of illness and coexisting conditions in patients with a longer hospital stay, factors that have been commonly associated with the traditional (non-SCC*mec* type IV) HA-MRSA infections. However, measures of severity of illness and coexisting conditions were not captured in this investigation. A third risk factor was a later year of culture collection; the likelihood of SCC*mec* type IV phenotype peaked in 2003. The rise of these isolates in our hospital may be from CA-MRSA isolates brought in from colonized persons from the community. CA-MRSA infections in Los Angeles County have rapidly become common and now exceed the frequency of those caused by CA–methicillin-susceptible *S. aureus* (*34*). Alternatively, the rise of SCC*mec* type IV isolates may be a result of spread throughout our hospital by the usual means of dissemination in a healthcare setting (e.g., hands of healthcare workers, contaminated environment) (*35*) or possibly by a combination of factors.

Exactly why the SCC*mec* type IV strains are successful in hospital settings such as ours and others (*20*) is unknown. Some evidence indicates that SCC*mec* type IV strains may be more “fit” than SCC*mec* types II/III that contain HA-MRSA isolates. Compared with methicillin-susceptible *S. aureus,* isolates containing SCC*me*c type II/III replicate more slowly in vitro (*36*). Okuma et al. found that CA-MRSA isolates that contain SCC*mec* type IV replicate more rapidly than these traditional HA-MRSA strains and argued that CA-MRSA may have enhanced ecologic fitness compared with SCC*mec* type II/III isolates, perhaps due simply to a shorter doubling time (*37*). Given the vulnerable population within the hospital setting, it is unclear how infections with isolates that contain SCC*mec* type IV will differ in symptoms and severity from those caused by traditional HA-MRSA isolates. On the basis of our study and other somewhat similar reports (*20*), concern is rising that USA300 strains may overtake the traditional HA-MRSA strains in many hospital and healthcare settings.

Our investigation had some limitations. First, the analysis was retrospective and thus it was not possible to prospectively identify patients with HA infections and compare them with patients with CA infections. Although, by means of a chart review of a subset of patients who were selected by the criteria of a MRSA culture obtained ≥72 hrs after admission, none of these infections fulfilled the CDC definition of a CA-MRSA infection (*31*).

A second limitation was that our case definition was based on phenotypic criteria because nonbloodstream isolates had been discarded and the SCC*mec* type could not be validated. Traditionally, most HA-MRSA isolates in the United States carry SCC*mec* type II (and to a lesser extent, SCC*mec* type III) that encodes resistance to β-lactam antimicrobial agents bleomycin, macrolide-lincosamide-streptogamin B, aminoglycosides, and spectinomycin (*38*). Gentamicin resistance occurs in most strains that carry the SCC*mec* type II element but is conferred by the *aac6′–aph2′′* gene elsewhere on the chromosome and is frequently carried by transposon Tn*4001* (*11*,*16*). Therefore, to select for isolates that did not confer a phenotype typical of healthcare-associated or non-SCC*mec* type IV–containing isolates, the SCC*mec* type IV phenotype was defined as isolates that were resistant to oxacillin and susceptible to gentamicin, clindamycin, and trimethoprim-sulfamethoxazole.

Some banked isolates did not grow, and in 1 isolate we could not detect an SCC*mec* element. Of note, stored isolates may lose their SCC*mec* elements over time (*39*), which may explain our findings. Nevertheless, over the 6-year observation period of our investigation, among isolates, the phenotype and genotype definition of SCC*mec* type were in agreement for >90% of isolates. Thus, we were able to validate our case definition of an HA-MRSA isolate with SCC*mec* type IV phenotype using both chart review and SCC*mec* typing.

A third limitation of our investigation was that we were able to recover only bloodstream isolates, a subset of strains that are small and potentially nonrepresentattive. Whether the relationship of phenotype to genotype is similar for bloodstream and nonbloodstream infections is unclear. A fourth limitation is that all of the patients were from 1 institution and, therefore, may only reflect local trends. However, as previously mentioned, reports of isolates associated with the CA-MRSA infections causing HA infections are growing (*17*–*20*).

In summary, we found that over a 5-year span, MRSA with a CA-MRSA phenotype has become the most common cause of HA-MRSA infections in our institution. This finding has important implications for MRSA epidemiology, infection control practices, and empiric antimicrobial drug selection.
